# Association of obesity with hyperuricaemia among HIV-positive patients on antiretroviral therapy in South-Western Uganda

**DOI:** 10.4102/ajlm.v14i1.2565

**Published:** 2025-02-19

**Authors:** Simon P. Rugera, Hope Mudondo, Jazira Tumusiime, Rahma Udu, Ritah Kiconco, Sylvia A. Lumumba, Charles N. Bagenda

**Affiliations:** 1 Department of Medical Laboratory Science, Faculty of Medicine, Mbarara University of Science and Technology, Mbarara, Uganda; 2 Department of Pure and Applied Sciences, School of Applied and Health Sciences, Technical University of Mombasa, Mombasa, Kenya; 3 Department of Biochemistry, School of Health Sciences, Soroti University, Soroti, Uganda; 4 Department of Medical Science, School of Applied and Health Sciences, Technical University of Mombasa, Mombasa, Kenya

**Keywords:** Hyperuricemia, obesity, human immunodeficiency virus, antiretroviral therapy, dolutegravir

## Abstract

**Background:**

Hyperuricaemia is a risk factor for gout and independently predicts hypertension, diabetes, and chronic kidney disease development. While elevated uric acid levels occur in HIV patients, and weight gain is linked to dolutegravir-based therapy, data on the obesity-hyperuricaemia relationship in this population remain limited.

**Objective:**

The objective of our study was to evaluate the association between obesity and hyperuricaemia among HIV-positive patients on antiretroviral therapy in South-Western Uganda.

**Methods:**

Between April 2024 and June 2024, this study conducted a secondary analysis of data on uric acid level and factors associated with obesity from a 2023 cross-sectional study of HIV-positive participants. We used logistic regression to assess the factors associated with hyperuricaemia, and receiver operating characteristic curve analysis to assess the predictive performance of body mass index for hyperuricaemia.

**Results:**

Among 328 participants, hyperuricaemia prevalence was 23.48% (95% confidence interval [CI]: 19.19–28.39%) higher in male participants (31.6%) than female participants (20.0%, *p* = 0.023). Overweight (adjusted odds ratio [aOR]: 2.01; 95% CI: 1.01–4.00; *p* = 0.046), obesity (aOR: 2.50; 95% CI: 1.09–5.73, *p* = 0.030), and male gender (aOR: 2.31; 95% CI: 1.07–5.01, *p* = 0.033) were significantly associated with hyperuricaemia.

**Conclusion:**

Our findings indicate a relationship between hyperuricaemia and obesity in HIV patients on antiretroviral therapy in Uganda. Nationwide studies using primary data are needed to better understand this relationship’s epidemiological spread.

**What this study adds:**

This study is the first to link obesity with hyperuricaemia among HIV-positive Ugandans on antiretroviral therapy, highlighting obesity as a key metabolic complication of HIV treatment.

## Introduction

Serum uric acid (SUA) is the end product of purine metabolism in humans.^1^ Almost two-thirds of SUA is produced endogenously, and the rest as a result of a diet abundant in purines.^2,3^ The kidneys excrete more than 70% of uric acid, with intestinal and biliary secretion accounting for a lesser amount.^3^ Hyperuricaemia and gout development most commonly result from abnormalities in SUA metabolism and its decreased excretion by the kidneys.^4^

The prevalence of hyperuricaemia has been rising rapidly, worldwide, in recent decades. Reports indicate that the prevalence of hyperuricaemia ranges from 0.1% to 10% globally, with an incidence of 0.3 to 6 cases per 1000 person years.^5^ Other data indicate that hyperuricaemia is prevalent not only in developed countries,^6,7^ but is also rising more frequently in low- and middle-income countries.^5,8,9^ Lifestyle factors such as physical exercise, obesity, high purine diet, and alcohol consumption have been highlighted as independent indicators of the emergence of hyperuricaemia.^10,11,12^ In addition, increased endogenous uric acid synthesis and exogenous protein consumption are associated with hyperuricaemia in obese individuals.^13^

Hyperuricaemia is a significant pathological condition that not only causes gout, but also acts as an independent risk factor for the onset of several other illnesses,^14^ such as diabetes mellitus,^15^ hypertension,^16,17^ chronic kidney disease,^18^ end-stage kidney disease,^19^ obesity,^20^ and metabolic syndrome,^21^ which are defined as a collection of risk factors for cardiovascular disease, including low levels of high-density lipoprotein cholesterol, hypertriglyceridaemia, central obesity, hypertension, and high glucose levels.^22^ Among these disorders, obesity has been found to be spreading quickly worldwide.^23^ According to the World Health Organization, 16% of adults were living with obesity in 2022.^24^ In addition to having a negative impact on a person’s health, obesity places a heavy burden on the healthcare system.^25^ Furthermore, it has been identified as a risk factor linked to several detrimental health outcomes, such as diabetes, hypertension, and high SUA levels.^6^ A number of studies on metabolic syndrome indicate that SUA is strongly associated with a number of indices, including waist circumference, body mass index (BMI), and dyslipidaemia.^20,26^ Consequently, hyperuricaemia is thought to be a prevalent lifestyle condition associated with obesity.^6,26^

Obesity in the last decade has been linked to various clinical conditions, including hyperuricaemia.^27^ Because obesity has detrimental metabolic effects that lower life expectancy, increase morbidity and mortality, and negatively impact quality of life, it poses a severe danger to public health and the expense of healthcare globally.^23^ Following World Health Organization recommendations in 2018, dolutegravir-based antiretroviral therapy (ART) was adopted as the first-line treatment for all people living with HIV in Uganda.^28^ Despite its benefits, recent literature indicates a strong association between the initiation on dolutegravir-based ART and subsequent weight gain and obesity.^29,30^ However, research about the relationship of obesity and SUA in the HIV patients receiving antiretroviral medication is scarce in our setting. Therefore, the aim of our study was to evaluate the prevalence of hyperuricaemia and its relationship with obesity among people with HIV on ART.

## Methods

### Ethical considerations

Ethical clearance was obtained from the Research Ethics Committee of Mbarara University of Science and Technology (reference no.: MUST-2023-1039) on 13 September 2023 for the cross-sectional study whose data set was used in the secondary data analysis of the current study. Further clearance to use the primary data for secondary data analysis was obtained from the Research Ethics Committee of Mbarara University of Science and Technology (reference no.: 36/05-17/MUST-2023-1039). In addition, the principal investigator and the research assistants of the primary study obtained informed written consent from each participant before enrollment into the study. Standard clinical procedures and regulations were followed, and privacy, anonymity, and confidentiality were protected during data collection and handling in the primary study. The participants were selected using simple random sampling and allotted identification numbers to replace names. The questionnaires were locked in a cabin accessed by only the principal investigator. The data set obtained from the primary study was used purposely for secondary data analysis based on the ethical clearance. All data were handled according to national and international data protection regulations, to ensure that the participants’ rights and privacy were respected.

### Study design, setting and population

This study was a secondary data analysis of a data set from a cross-sectional study conducted among HIV-positive participants on ART from 21 September 2023 to 21 October 2023 at Mbarara Municipal Council Health Centre IV, South-Western Uganda. The title of the cross-sectional study whose data set has been used for secondary analysis was ‘Correlation of serum uric acid/serum creatinine ratio with estimated glomerular filtration rate in predicting kidney dysfunction among people living with HIV at Mbarara Municipal Council Health Centre IV, Uganda’.^31^ The source population of the cross-sectional study was all adult HIV-positive patients on ART, while those systematically selected HIV-positive patients attending Mbarara Municipal Council Health Centre IV ART Clinic during the period of data collection were the study population.

### Sample size and sampling technique

The primary study used the Kish and Lesly formula to calculate the sample size. The sample size of 328 study participants was established in the cross-sectional study whose major variable was kidney dysfunction. A systematic sampling technique was used to select adult HIV-positive patients receiving ART in the clinic to participate in the cross-sectional study.

### Eligibility criteria

The cross-sectional study included all adult HIV-positive patients who were aged 18 years and above, and who had been on ART for at least 12 months, using a systematic selection procedure. However, the study excluded adult HIV-positive patients receiving ART who had known kidney-related illnesses, as well as those who were unable to communicate.

### Variables and equipment

The major outcome variable in the cross-sectional study was kidney dysfunction, which was defined as an estimated glomerular filtration rate (eGFR) < 90 mL/min, and the main exposure variable was SUA/creatinine ratio. In this secondary data analysis study, the main outcome variable was hyperuricaemia. Body mass index is categorised into < 25 kg/m^2^ (normal weight), 25 kg/m^2^ – 29.9 kg/m^2^ (overweight), and ≥ 30 kg/m^2^ (obesity), and was the major independent variable. The other independent variables that were explored in the data set for their relationship with hyperuricaemia included social demographics (gender, marital status, education level, religion, employment status, residence), smoking status, alcohol consumption status, blood pressure (BP), physical activity, family history of hypertension, family history of kidney disease, family history of cardiovascular disease, duration on ART, viral load, eGFR, and creatinine level.

The information on social demographics, family background for chronic diseases (hypertension, kidney disease, cardiovascular diseases), and behavioural factors was collected by trained research assistants who directly interviewed study participants in the local language using a semi-structured questionnaire. The trained research assistants also reviewed the medical records of the consented study participants to obtain data regarding the duration of ART, recent viral load results, and medication. The measurement of height and weight that were required for the calculation of BMI, waist/hip circumferences needed to calculate waist-to-hip ratio, and BP were also performed by the two research assistants. A digital sphygmomanometer was used to take BP measurements. Two BP readings were recorded at a 5 min interval, and their mean value was taken as the BP. The weight and height of the participants were measured using a portable weight and height scale.

Approximately 4 mL of venous blood was collected from each study participant, using red top (serum) vacutainer vacuum tubes, by the principal investigator and a laboratory assistant attached to the ART clinic. Serum was separated by centrifugation at 3000 rpm for 4 min within a period not more than 1 h after blood collection. The serum concentrations of uric acid and creatinine were measured using the cobas^©^ 111 analyser (Roche Diagnostics International, Rotkreuz, Switzerland) at the Clinical Chemistry department laboratory at Mbarara City Health Centre IV. This instrument is designed to perform spectrophotometric measurements of the analyte level at a specific set of wavelengths using specific reagents. The analyser automatically performs all reagent and sample pipetting, incubation, photometric measurements, and calculations. Calibration and quality control of this chemistry analyser were performed before the samples were run. The manufacturer’s instructions for the machine and the reagents were strictly followed. In order to contribute to the quality control process, 10% of the samples were run at Mbarara Regional Referral Hospital for comparison, using the HumaStar 200 (Human Diagnostics Worldwide, Wiesbaden, Germany). There was no statistical difference between the results from both laboratories.

### Statistical analysis

Data were analyzed using STATA 17.0 (StataCorp LLC, College Station, Texas, United States). Creatinine and SUA:creatinine ratio were tested for normality using the Shapiro-Wilk normality test; neither was normally distributed. They were therefore summarised using the median with interquartile range, and their medians were compared between the participants with hyperuricaemia and those without hyperuricaemia using the Wilcoxon-Rank Sum test. Uric acid levels were categorised into hyperuricaemia present (uric acid > 7 mg/dL in men and uric acid > 6 mg/dL in women) and hyperuricaemia absent. The eGFR rate was also categorised into kidney dysfunction present (eGFR < 90 mL/min) and kidney dysfunction absent (eGFR ≥ 90 mL/min). A categorical variable, hypertension (normal BP = systolic BP < 140 mmHg and diastolic BP < 90 mmHg; systolic hypertension only = systolic BP ≥ 140 mmHg and diastolic BP < 90 mmHg; diastolic hypertension only = systolic BP < 140 mmHg and diastolic BP ≥ 90 mmHg; and both systolic and diastolic hypertension = systolic BP ≥ 140 mmHg and diastolic BP ≥ 90 mmHg), was generated from both continuous systolic and diastolic BP variables. All categorical variables were summarised using frequencies and percentages.

The prevalence of hyperuricaemia, together with its 95% confidence interval (CI), was obtained by dividing the number of participants with hyperuricaemia by the total number of participants that were recruited in the study (sample size), expressed as a percentage.

To assess the association between categorised BMI and hyperuricaemia, we used logistic regression. Hyperuricaemia was the binary dependent variable. All independent variables, including the main independent variable (categorised BMI) at the bivariate level, were compared with hyperuricaemia (uric acid levels that were dichotomised into high and normal levels). The associations were determined using odds ratios (OR) together with their 95% CI, and the statistically significant ORs were indicated by a *p*-value of < 0.2 at the bivariate level. The variables that were clinically and/or statistically significant at this level were also included in the multivariable model. The Hosmer-Lemeshow test was used to test the suitability of the final model in predicting the outcome variable: hyperuricaemia. In the final multivariable model, associations were considered significant at a *p*-value ≤ 0.05.

To assess the predictive performance of BMI for hyperuricaemia, we used a receiver operating characteristic curve. Predictive power was evaluated using the area under the curve (AUC) and its 95% CI; a value near to 1 indicates better performance and a 95% CI that included 0.5 indicates a non-significant ability to discriminate between those with hyperuricaemia and those without hyperuricaemia. We used the Youden index to estimate the optimal cutoff point of BMI for hyperuricaemia prediction at maximum sensitivity and specificity.

## Results

### Characteristics of the study participants

A total of 328 participants were recruited in this study, of which 97.3% were on dolutegravir-based ART. The median age of the study participants was 38 years, with an interquartile range of 31 years – 47 years. The majority of the study participants were women (230/328; 70.1%), married or cohabiting (185/328; 56.6%), had completed the primary level of education (179/328; 54.6%), were Protestant by religion (155/328; 47.3%), and unemployed (173/328; 52.7%) (Table 1).

**TABLE 1:** Characteristics of study participants stratified by hyperuricaemia status among HIV-positive patients in South-Western Uganda, April 2024 to June 2024.

Variable	*n*	%	Median	IQR	Hyperuricaemia								*p*
					Absent				Present				
					*n*	%	Median	IQR	*n*	%	Median	IQR	
**Total**	-	-	-	-	251	76.52	-	-	77	23.48	-	-	-
**Age (years)**	-	-	38	31–47	-	-	38	30–46	-	-	40	33–50	0.077
**Gender**	-	-	-	-	-	-	-	-	-	-	-	-	0.023*
Male	98	29.9	-	-	67	68.4	-	-	31	31.6	-	-	-
Female	230	70.1	-	-	184	80.0	-	-	46	20.0	-	-	-
**Marital status**	-	-	-	-	-	-	-	-	-	-	-	-	0.97
Single	79	24.1	-	-	60	75.9	-	-	19	24.1	-	-	-
Married/cohabiting	185	56.6	-	-	142	76.8	-	-	43	23.2	-	-	-
Separated/divorced	63	19.3	-	-	49	77.8	-	-	14	22.2	-	-	-
**Education**	-	-	-	-	-	-	-	-	-	-	-	-	0.75
No education	34	10.4	-	-	27	79.4	-	-	7	20.6	-	-	-
Primary	179	54.6	-	-	139	77.7	-	-	40	22.3	-	-	-
Secondary	93	28.3	-	-	70	75.3	-	-	23	24.7	-	-	-
Tertiary	22	6.7	-	-	15	68.2	-	-	7	31.8	-	-	-
**Religion**	-	-	-	-	-	-	-	-	-	-	-	-	0.79
Catholic	117	35.7	-	-	90	76.9	-	-	27	23.1	-	-	-
Protestant	155	47.3	-	-	121	78.1	-	-	34	21.9	-	-	-
Moslem	29	8.8	-	-	21	72.4	-	-	8	27.6	-	-	-
Others	27	8.2	-	-	19	70.4	-	-	8	29.6	-	-	-
**Employment**	-	-	-	-	-	-	-	-	-	-	-	-	0.047*
Unemployed	173	52.7	-	-	140	80.9	-	-	33	19.1	-	-	-
Employed	155	47.3	-	-	111	71.6	-	-	44	28.4	-	-	-
**Smoking**	-	-	-	-	-	-	-	-	-	-	-	-	0.87
Non-smoker	302	92.6	-	-	231	76.5	-	-	71	23.5	-	-	-
Ever smoked	24	7.4	-	-	18	75.0	-	-	6	25.0	-	-	-
**Residence**	-	-	-	-	-	-	-	-	-	-	-	-	0.41
Rural	115	35.1	-	-	85	73.9	-	-	30	26.1	-	-	-
Urban	213	64.9	-	-	166	77.9	-	-	47()	22.1	-	-	-
**Alcohol status**	-	-	-	-	-	-	-	-	-	-	-	-	0.13
Never consumed	224	68.5	-	-	177	79.0	-	-	47	21.0	-	-	-
Current consumer	86	26.3	-	-	59	68.6	-	-	27	31.4	-	-	-
Past consumer	17	5.2	-	-	14	82.4	-	-	3	17.6	-	-	-
**Physical activity**	-	-	-	-	-	-	-	-	-	-	-	-	0.83
No	45	13.7	-	-	35	77.8	-	-	10	22.2	-	-	-
Yes	283	86.3	-	-	216	76.3	-	-	67	23.7	-	-	-
**Sleep duration**	-	-	8	8–9	-	-	8	7–9	-	-	8	8–9	0.12
**Hypertension**	-	-	-	-	-	-	-	-	-	-	-	-	0.74
Normal	211	64.3	-	-	165	78.2	-	-	46	21.8	-	-	-
Systolic	14	4.3	-	-	11	78.6	-	-	3	21.4	-	-	-
Diastolic	53	16.2	-	-	39	73.6	-	-	14	26.4	-	-	-
Both systolic and diastolic	50	15.2	-	-	36	72.0	-	-	14	28.0	-	-	-
**Body mass index (kg/m^2^)**	-	-	-	-	-	-	-	-	-	-	-	-	0.17
< 25	174	53.4	-	-	140	80.5	-	-	34	19.5	-	-	-
25–29.9	94	28.8	-	-	68	72.3	-	-	26	27.7	-	-	-
> 30	58	17.8	-	-	41	70.7	-	-	17	29.3	-	-	-
**Family history hypertension**	-	-	-	-	-	-	-	-	-	-	-	-	0.73
No	250	76.5	-	-	190	76.0	-	-	60	24.0	-	-	-
Yes	77	23.5	-	-	60	77.9	-	-	17	22.1	-	-	-
**Family of chronic kidney disease**	-	-	-	-	-	-	-	-	-	-	-	-	0.98
No	306	93.6	-	-	234	76.5	-	-	72	23.5	-	-	-
Yes	21	6.4	-	-	16	76.2	-	-	5	23.8	-	-	-
**Family history of cardiovascular disease**	-	-	-	-	-	-	-	-	-	-	-	-	0.70
No	307	93.9	-	-	234	76.2	-	-	73	23.8	-	-	-
Yes	20	6.1	-	-	16	80.0	-	-	4	20.0	-	-	-
**Duration of antiretroviral therapy (years)**	-	-	-	-	-	-	-	-	-	-	-	-	0.61
< 2	23	7.1	-	-	19	82.6	-	-	4	17.4	-	-	-
> 2	301	92.9	-	-	228	75.7	-	-	73	24.3	-	-	-
**Viral load**	-	-	-	-	-	-	-	-	-	-	-	-	0.13
Suppressed	315	96.3	-	-	243	77.1	-	-	72	22.9	-	-	-
Non-suppressed	12	3.7	-	-	7	58.3	-	-	5	41.7	-	-	-
**Estimated glomerular filtration rate (< 60 mL/min)**	-	-	-	-	-	-	-	-	-	-	-	-	0.43*
Absent	301	91.8	-	-	232	77.1	-	-	69	22.9	-	-	-
Present	27	8.2	-	-	19	70.4	-	-	8	29.6	-	-	-
**Creatinine (µmol/L)**	-	-	78.75	67.4–89.75	-	-	78.6	66.70–88.90	-	-	79.3	690.3–96.50	0.34
**Serum uric acid/creatinine ratio**	-	-	3.82	3.05–4.73	-	-	3.53	2.93–4.17	-	-	5.46	4.65–6.55	< 0.001*

IQR, interquartile range; CI, confidence interval.

* , statistically significant.

### Prevalence of hyperuricaemia

**FIGURE 1:**
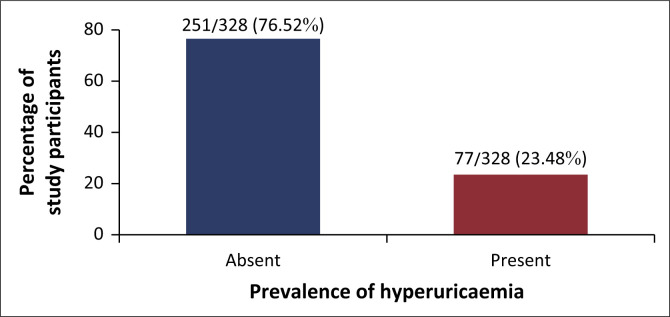
A bar graph showing prevalence of hyperuricaemia among HIV-positive patients in South-Western Uganda, April 2024 to June 2024.

Out of the 328 study participants, 77 had hyperuricaemia, giving an overall prevalence of 23.48% with a 95% CI of 19.19% to 28.39%, as indicated in Figure 1. The prevalence of hyperuricaemia was significantly higher among the male participants (*n* = 31; 31.6%) than the female participants (*n* = 46; 20.0%), *p*-value 0.023. We also observed a significantly higher prevalence of hyperuricaemia among employed participants (*n* = 44; 28.4%) in comparison to the unemployed (*n* = 33; 19.1%), *p*-value 0.047 (Table 1).

### Association between obesity and hyperuricaemia

Overweight (adjusted OR: 2.01; 95% CI: 1.01–4.00; *p* = 0.046), obesity (adjusted OR: 2.50; 95% CI: 1.09–5.73, *p* = 0.030), and male gender (adjusted OR: 2.31; 95% CI: 1.07–5.01; *p* = 0.033) were significantly associated with the prevalence of hyperuricaemia among HIV-positive patients (Table 2).

**TABLE 2:** Factors associated with hyperuricaemia among HIV-positive patients in South-Western Uganda, April 2024 to June 2024.

Variable	Bivariate analysis			Multivariate analysis		
	cOR	95% CI	*p*	aOR	95% CI	*p*
**Age**	1.02	0.99 – 1.04	0.150	1.02	0.99 – 1.05	0.168
**Gender**						
Female	1.00	-	-	1.00	-	-
Male	1.85	1.08 – 3.16	0.024*	2.31	1.07 – 5.01	0.033*
**Marital status**						
Single	1.00	-	-	-	-	-
Married or cohabiting	0.96	0.52 – 1.78	0.887	-	-	-
Separated or divorced	0.90	0.41 – 1.98	0.798	-	-	-
**Education**						
No education	1.00	-	-	-	-	-
Primary	1.11	0.45 – 2.74	0.821	-	-	-
Secondary	1.27	0.49 – 3.30	0.627	-	-	-
Tertiary	1.80	0.53 – 6.12	0.346	-	-	-
**Religion**						
Catholic	1.00	-	-	-	-	-
Protestant	0.94	0.53 – 1.66	0.823	-	-	-
Moslem	1.27	0.51 – 3.19	0.611	-	-	-
Others	1.40	0.55 – 3.56	0.476	-	-	-
**Employment**						
Unemployed	1.00	-	-	1.00	-	-
Employed	1.68	1.01 – 2.82	0.048*	1.65	0.93 – 2.93	0.086
**Smoking**						
Non-smoker	1.00	-	-	1.00	-	-
Ever smoked	1.08	0.41 – 2.84	0.869	0.67	0.22 – 2.03	0.480
**Residence**						
Rural	1.00	-	-	-	-	-
Urban	0.80	0.47 – 1.35	0.413	-	-	-
**Alcohol status**						
Never consumed	1.00	-	-	1.00	-	-
Current consumer	1.72	0.99 – 3.01	0.056	1.42	0.74 – 2.74	0.295
Past consumer	0.81	0.22 – 2.93	0.744	0.82	0.19 – 3.45	0.785
**Physical activity**						
No	1.00	-	-	1.00	-	-
Yes	1.09	0.51 – 2.31	0.831	0.92	0.41 – 2.08	0.837
Sleep duration	1.09	0.90 – 1.33	0.361	1.15	0.93 – 1.42	0.194
**Hypertension**						
Normal	1.00	-	-	1.00	-	-
Systolic	0.98	0.26 – 3.65	0.974	0.62	0.15 – 2.55	0.504
Diastolic	1.29	0.64 – 2.57	0.474	1.29	0.61 – 2.75	0.503
Both systolic and diastolic	1.39	0.69 – 2.80	0.350	1.23	0.58 – 2.65	0.589
**Body mass index (kg/m^2^)**						
< 25	1.00	-	-	1.00	-	-
25–29.9	1.57	0.88 – 2.83	0.130	2.01	1.01 – 4.00	0.046*
> 30	1.71	0.87 – 3.36	0.122	2.50	1.09 – 5.73	0.030*
**Family history of hypertension**						
No	1.00	-	-	1.00	-	-
Yes	0.89	0.49 – 1.65	0.728	0.85	0.41 – 1.76	0.660
**Family history of chronic kidney disease**						
No	1.00	-	-	1.00	-	-
Yes	1.02	0.36 – 2.87	0.977	1.03	0.33 – 3.22	0.962
**Family history of cardiovascular disease**						
No	1.00	-	-	1.00	-	-
Yes	0.80	0.26 – 2.47	0.700	1.01	0.28 – 3.68	0.987
**Duration of antiretroviral therapy (years)**						
< 2	1.00	-	-	1.00	-	-
> 2	1.52	0.50 – 4.61	0.459	1.61	0.48 – 5.44	0.442
**Viral load**						
Suppressed	1.00	-	-	1.00	-	-
Non-suppressed	2.41	0.74 – 7.82	0.143	3.36	0.86 – 13.20	0.086
**Estimated glomerular filtration rate (< 60 mL/min)**						
Absent	1.00	-	-	1.00	-	-
Present	1.42	0.59 – 3.37	0.433	1.23	0.35 – 4.36	0.751
**Creatinine (µmol/L)**	1.01	0.99 – 1.02	0.234	0.99	0.98 – 1.01	0.838

cOR; corrected odds ratio; aOR, adjusted odds ratio; CI, confidence interval.

* , statistically significant.

### Predictive performance of body mass index for hyperuricaemia

**FIGURE 2:**
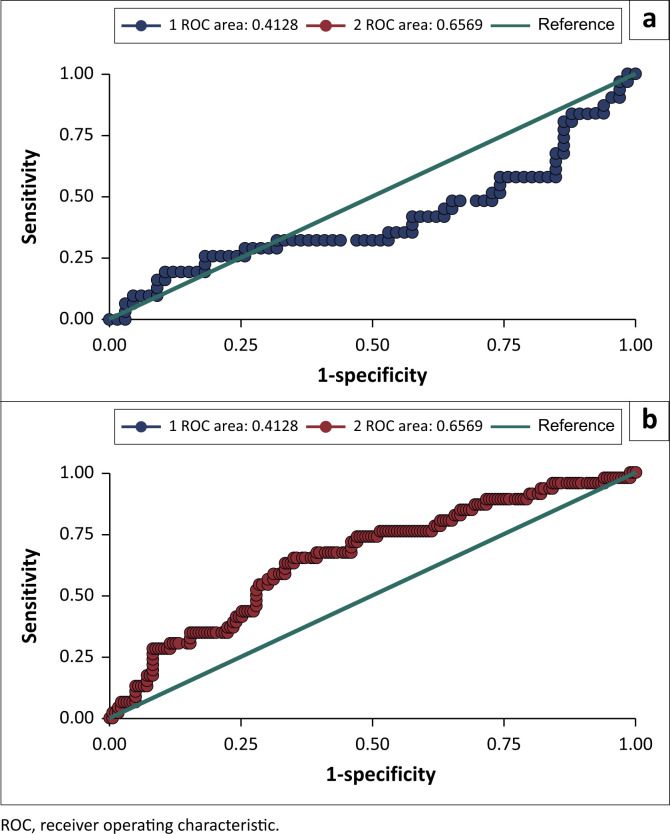
The receiver operating characteristic curves of the body mass index for separating participants with and without hyperuricaemia among (a) male and (b) female HIV-positive patients in South-Western Uganda, April 2024 to June 2024.

Body mass index did not show a significant predictive power for identifying hyperuricaemia, as the 95% CI includes a null value of 0.5, which randomly classifies participants in the two groups (Table 3). However, a significant difference in the predictive performance of BMI for hyperuricaemia was noted between male participants (AUC = 0.4128) and female participants (AUC = 0.657), *p*-value 0.0029 (Table 3 and Figure 2). Body mass index showed a significant predictive performance for hyperuricaemia among female participants (AUC: 0.657; 95% CI: 0.568–0.746), while the predictive performance was not significant among male participants (AUC = 0.4128; 95% CI: 0.279–0.546). At an optimal cutoff point of ≥ 26.806 kg/m^2^, BMI can significantly separate female participants with or without hyperuricaemia at both a sensitivity and specificity of 65% (Table 3).

**TABLE 3:** Predictive performance of body mass index for hyperuricaemia among HIV-positive patients in South-Western Uganda, April 2024 to June 2024.

Category	AUC	95% CI	Optimal cutoff point	Sensitivity (%)	Specificity (%)	Youden index
Overall	0.543	0.463 – 0.623	≥ 26.805	47	71	0.178
Male	0.413	0.279 – 0.546	≥ 27.137	19	89	0.087
Female	0.657	0.568 – 0.746	≥ 26.806	65	65	0.302

Note: H0: area (Male) = area (Female); *p*-value = 0.0029. The *p*-value (0.0029) indicates that there is a statistically significant difference between the ‘area’ for men and women. Therefore, the null hypothesis (H0) is rejected.

AUC, area under the curve; CI, confidence interval.

## Discussion

Our findings indicate for the first time the relationship between obesity and hyperuricaemia in Uganda. In our study, the overall prevalence of hyperuricaemia was 23.48% (95% CI: 19.19–28.39), which falls within the worldwide prevalence, which is reported to range from 2.6% to 36.0% in different populations by a study carried out in Bangkok in 2011.^32^ Our overall prevalence is also in agreement with the hyperuricaemia prevalence of 25.2% reported among HIV-positive patients receiving stable highly active ART in Perugia in 2018.^33^ However, our study prevalence is significantly lower than a 2021 study on South Indian adult patients with stable coronary artery disease (46.5%).^34^ Limited studies done in Africa have tried to establish the prevalence of hyperuricaemia among HIV patients. A study done in Zimbabwe in 2017 found a prevalence of 13.0%, which was lower than our finding.^35^ Since literature is scanty, we had to compare our results with dissimilar studies that did not deal with HIV patients. Our results are in agreement with the 20.5% prevalence reported among adults in Nigeria in 2016,^36^ and the 28.0% prevalence among outpatients at Bafoussam Regional Hospital in Gabon in 2024,^37^ but are in disagreement with the prevalence of 31.0% reported by a cross-sectional study conducted in Ethiopia among adult staff members of a public health institute.^34^ These discrepancies may be because of differences in ethnic background and study populations.

A significantly higher prevalence of hyperuricaemia was observed among male participants than female participants, as was also reflected in previous studies.^38,39,40^ Our study notes a significant association between male gender and the presence of hyperuricaemia among HIV patients on ART. This association is in agreement with the findings of a study conducted in Kenya in 2018 among university workers that found that hyperuricaemia was threefold higher in male participants (adjusted OR 2.938; 95% CI, 1.909–4.522, *p* < 0.01) than female participants.^39^ A related study in Ethiopia in 2021, though in a different study population, also observed a significant association between male gender and hyperuricaemia.^34^

Our findings in respect to the odds of hyperuricaemia (which were 2.01 times higher in overweight participants and 2.5 times higher in obese participants than in participants with a BMI < 25 kg/m^2^) were consistent with results from a Zimbabwean study in 2017 that showed a significant association between hyperuricaemia and a BMI (an indicator of obesity) > 30 kg/m^2^,^35^ and a cross-sectional study among male adults in Nigeria in 2015.^41^ These findings were also in agreement with a study conducted on Bangladeshi adults in 2018 that found that even after adjusting for lipid profile, age and sex, the prevalence of obesity increased progressively across the SUA quartiles. The SUA levels were also closely and independently associated with obesity.^4^ Another recent nationwide study in China done in 2024 also documented a significant association between obesity indices, including BMI and risk of hyperuricaemia.^42^ Also, in line with our findings, numerous studies have demonstrated a positive association between SUA and obesity in various populations. For instance, a 10-year follow-up research study in 2007 revealed that, across all race-sex-groups, rising SUA levels were substantially correlated with higher BMI.^43^

Studies from different ethnicities also found a positive relationship between hyperuricaemia and obesity. For example, Tanaka et al. established that, after correcting for genetic and environmental variables in both genders, there was a significant correlation between BMI and SUA levels in Japanese adult twins in 2015.^26^ Another study by Wang et al. reported that, among healthy people in the Chinese province of Jiangsu in 2014, there was a positive correlation between BMI and SUA levels.^20^ More recent research has also shown a strong positive correlation between SUA levels and obesity in the Chinese population in 2017 and 2018,^44,45,46^ Japan in 2006,^47^ India in 2014,^6,48^ Pakistan in 2009,^49^ Iraq in 2015,^50^ and the United States in 2004.^51^

Although earlier research has shown a positive association between SUA levels and obesity, the exact mechanism by which obesity causes an increase in uric acid is not yet thoroughly understood. The relationship between obesity and SUA may be explained using two factors: overproduction and poor renal excretion. An investigation carried out on a Japanese population who had visceral fat obesity revealed that elevated uric acid levels are significantly impacted by its excessive synthesis, with decreased excretion and clearance of urates in the urine.^52^ Furthermore, the buildup of visceral fat causes an increased flow of free fatty acids from plasma into the liver and hepatic portal vein, which in turn promotes triglyceride synthesis and subsequently increases the production of uric acid by activating the uric acid synthesis pathway.^53,54^

Our study also indicates a significantly higher predictive performance of BMI for hyperuricaemia in female participants (AUC = 0.657) than in male participants (AUC = 0.513), *p*-value 0.0029. Our finding is consistent with findings from a large Taiwanese population study done in 2023 that also found BMI to have a higher predictive power for hyperuricaemia in female participants (AUC = 0.728) than in male participants (AUC = 0.655).^55^

### Limitations

The study is a secondary data analysis; hence, we had limited control over the data collection process. As the study used a data set from a cross-sectional study, we could not establish the cause-effect relationship. The generalisability of our findings is limited to HIV-positive patients on dolutegravir-based ART, and the results may not apply to those on different ART regimens or other populations.

### Conclusion

Our findings are the first of their kind to indicate a relationship between hyperuricaemia and obesity in HIV patients on ART in Uganda. The high prevalence of hyperuricaemia in these patients requires the attention of policymakers with regard to the institution of routine measurement of SUA in obese and overweight people to prevent hyperuricaemia and its related complications. A study that does not rely on secondary data should be carried out country wide to further enhance our understanding of the epidemiological spread of this relationship.
